# The use of atypical antipsychotic medications in the treatment of children and adolescents with avoidant/restrictive food intake disorder

**DOI:** 10.1007/s00787-025-02713-w

**Published:** 2025-04-24

**Authors:** Mariela Mosheva, Yaron Sela, Shani Arad-Rubinshtein, Yaffa Serur, Ganit Omer, Nimrod Hertz-Palmor, Doron Gothelf, Daniel Stein

**Affiliations:** 1 Child Psychiatry Division, The Edmond and Lily Safra Children’s Hospital, Sheba Medical Center, Tel Hashomer, 5262000 Israel; 2 The Faculty of Medical & Health Sciences, Tel Aviv University, Tel Aviv, Israel; 3 The Research Center for Internet Psychology, Reichman University, Herzliya, Israel; 4 Sagol School of Neuroscience, Tel Aviv University, Tel Aviv, Israel

**Keywords:** ARFID, Atypical antipsychotic medications, Body mass index, Height, Weight

## Abstract

**Introduction:**

Avoidant restrictive food intake disorder (ARFID) is a childhood feeding and eating disorder often associated with marked physical and psychosocial impairment.

**Objective:**

We assessed the use of atypical antipsychotic (AAP) medications (mostly risperidone) in promoting weight and height gain in children with ARFID.

**Methods:**

The computerized medical records of 21 children with ARFID receiving AAPs in one center in Israel were retrospectively reviewed. Fourteen children received AAPs after 6.30 ± 0.75 months of no weight gain with treatment as usual (either group or individual cognitive behavioral therapy); seven children were admitted to our clinic receiving AAPs in previous facilities because of lack of weight gain. All were followed-up for 18 months. Weight and height were extracted from the medial records at eight time points.

**Results:**

A significant increase was found in weight, height, and body mass index (BMI) over 18 months of treatment with AAPs (Δweight: 9.66 ± 9.24 kg, *p* < 0.001; Δheight: 10.23 ± 11.54 cm, *p* < 0.001; ΔBMI = 2.55 ± 1.53 kg/m^2^; *p* < 0.001). Weight increased significantly for both sexes, while height increased significantly only for boys. Patients with both low and high baseline BMI percentiles gained weight, while mean height increased significantly over time only for children with low BMI percentile. The use of a retrospective clinical global impression scale indicated a marked improvement over time. Adverse effects were minimal, and no patients discontinued AAPs due to adverse events.

**Conclusion:**

The addition of AAPs for a period of 18 months may be safe and effective in increasing weight and height in children with ARFID.

**Supplementary Information:**

The online version contains supplementary material available at 10.1007/s00787-025-02713-w.

## Introduction

Avoidant/restrictive food intake disorder (ARFID) is a relatively new psychiatric diagnosis, appearing as a separate diagnosis for the first time in the 5th edition of the Diagnostic and Statistical Manual of Mental Disorders (DSM-5) [1]. It is also included currently in the 11th Revision of the World Health Organization’s International Classification for Diseases [(ICD-11)]. ARFID consists of a varied presentation of persistent restricting feeding and eating disturbances, resulting in nutritional deficiencies (and low weight in some although not all of the patients), failure to meet nutritional and/or energy needs, and related psychosocial disturbances [1]. ARFID is considered a complex, severe, and clinically challenging childhood-onset eating and feeding disorder potentially leading to considerable physiological and psychological morbidity in affected individuals [2]. It is of note that the symptoms of avoidance and restrictive eating in ARFID are unrelated to intentional weight loss, dieting behaviors, and body image disturbances [3]. Last, while ARFID usually starts during childhood, it may continue throughout the entire lifespan [4].

The DSM-5 [1] describes three primary ARFID subtypes including sensory sensitivity to the taste, smell or texture of food, lack of interest in food or eating, and fear of aversive consequences (e.g., chocking or vomiting). Many patients present with a combination of more than one subtype [5]. This mixed subtype is usually the most prevalent one, followed by sensory sensitivity, lack of interest, and fear of aversive consequences, respectively [5]. The four ARFID presentation groups, while presenting many similarities, may differ on core ARFID criteria, symptom trajectory and illness duration, mood and medical comorbidities, age, gender, and parent-reported symptoms of psychopathology [6]. Usually the lack of interest subtype is associated with the highest risk of weight reduction and low weight, and the sensory sensitivity subtype with the lowest [5, 6].

Currently, there is limited data on the incidence and prevalence of ARFID in the general population [7, 8]. Large-scale community studies have found that 3.2-15.5% of children may report symptoms of ARFID [9–12]. In eating disorder (ED) care clinics, the prevalence of ARFID is higher, ranging from 5 to 22% [13–17].

To date, most treatment studies in ARFID are case studies [4], including cognitive behavioral therapy (CBT) [18–20], or family-based therapy (FBT) [21, 22]. Recently, several CBT clinical guidelines and manuals have been developed for the treatment of ARFID [23–27]. A multi-professional team is required to effectively address ARFID treatment [28].

Data on the outcome and prognosis of ARFID are limited due to its recent classification as a separate entity. Weight gain achieved in many, although not all, cases [4, 29], has been found to be maintained at 30 months follow-up [30]. Nevertheless, some children with ARFID require inpatient treatment [31] or partial hospitalization [30] to induce weight gain. A small-scale study (*n* = 19) found that 26.3% of children with ARFID continue to meet diagnostic criteria after a mean follow-up of 15.4 years [32]. Another study of children (mean age 2 years) diagnosed with infantile anorexia (likely ARFID) found that 61% continue to exhibit moderate to severe malnutrition at 11 years of age [33].

The lack of favorable outcome of ARFID in some cases following psychological treatments calls for the addition of other potential treatment modalities. One such option is the use of atypical antipsychotic (AAP) medications, because of their potential to increase weight and reduce anxiety [34, 35]. There is only limited data on the use of AAPs for ARFID because of the concern of possible influences of these medications on the developing brain in young ages [36], and because of the reluctance of families and professionals to administer medications at younger ages [28, 37]. Nonetheless, a few case studies have explored the use of AAP medications to facilitate eating and increase weight gain in children with ARFID. A brief report of nine children with ARFID has found that olanzapine may facilitate eating, promote appetite, increase weight, and reduce anxiety and depressive symptoms [38]. Similar improvements have been reported in another case series describing six children with ARFID and comorbid anxiety, treated with a combination of family therapy and olanzapine, alongside other medications, including cyproheptadine [22]. Another case report has shown the beneficial effect of aripiprazole in reducing anxiety and facilitating eating in individuals with ARFID-related phagophobia [39]. Last, mirtazapine and buspirone have been found to relieve anxiety associated with choking and/or vomiting, increase appetite, and facilitate weight gain in youth with ARFID [40–42].

These findings initiated our attempt to evaluate the effectiveness of AAPs in promoting weight and height gain in children and adolescents with ARFID using a retrospective chart design. Risperidone was chosen as a first choice, because it is the only AAP allowed by the Israel Ministry of Health for treatment in pediatric psychiatric populations under the age of 18, and because it is the only AAP available in Israel in liquid form, which is beneficial for young children with ARFID who struggle with pill swallowing. Other AAPs can be given only according to special permission because of ineffectiveness or adverse effects of risperidone. We hypothesized that the addition of AAPs would significantly increase weight and promote linear growth and reduce the severity of the ARFID symptoms. Additionally, we anticipated that adverse symptoms following AAP treatment would be mild, and the medications would be well-tolerated by most of the participants.

## Methods

### Participants

The study retrospectively analyzed the computerized medical charts of 21 children diagnosed with ARFID treated with AAP medications. It was carried out in the Child and Adolescent Eating Disorder Outpatient Clinic at the Pediatric Psychosomatic Department, Safra Children’s Hospital, Sheba Medical Center, Tel Hashomer, Israel, between 01/01/2017 and 30/03/2021. During that time, 180 children were treated in our clinic for ARFID. The other 159 patients not included in the study did not require AAPs (the majority), their parents did not agree to AAP use if required, or they did not fulfil the required inclusion criteria.

Eligibility for the study included children with ARFID who did not gain weight with psychological therapy for at least six consecutive months (*n* = 14) or if they were admitted to our clinic from another facility due to their failure to gain weight and were already treated with AAPs when admitted to our facility (*n* = 7). These children began treatment with risperidone by their local psychiatric caregiver because of not gaining weight. They were referred to our clinic, being the only specialized clinic in Israel at that time, usually within a few weeks. We had no information as to the exact duration of risperidone treatment in these children before entering our study, but it was short. We considered their weight at the first assessment in our facility as Time 0., i.e., the time of the beginning of our treatment protocol with AAPs.

Children were excluded from the study if any the following conditions were met: (1) diagnosis of a comorbid medical disorder with the potential to influence feeding, eating, and weight (e.g., achalasia, thyroid dysfunction, or type I diabetes mellitus), (2) presence of eating and feeding difficulties due to other psychiatric disorders (e.g., anorexia nervosa, major depression), and (3) comorbid diagnosis of schizophrenic spectrum disorders, autistic spectrum disorders, or any neuropsychiatric disorder (These children were treated in our facility but were not included in the study as their treatment was highly difficult because of their primary diagnosis, requiring often specifically modified interventions).

Before enrolment, a detailed explanation has been provided by the treating child and adolescent psychiatrist about the reasons for recommending the addition of the AAP after there was no weight gain for six months with ARFID-geared psychological intervention for 14 patients, or for the continuation of the AAP in the 7 children already receiving it on their admission to our clinic, as well as about the benefits and adverse effects of this treatment. The parents have been informed that although this treatment is known for other pediatric psychiatric disorders, it has been infrequently used in ARFID. The treatment is offered because of its adverse effect of increasing weight but also because of its anxiolytic effect. Other adverse effects may appear, particularly drowsiness, fatigue and cognitive impairment, but they are self-limiting if the medication is discontinued. The parents and children have been informed that they may discontinue this treatment whenever they wished to do so and continue with the other treatment modalities used in our facility.

The protocol of this retroactive chart review design was approved by Internal Review board of the Sheba Medical Center (8102-21-SMC). Informed consent was waived because of the retrospective nature of the study, i.e., review of electronic medical records. The study was anonymous, and no names were provided.

## Assessment

### Diagnosis of ARFID and comorbid psychiatric disorders

Children considered of having ARFID are typically referred to our clinic by community providers, including pediatricians, clinical nutritionists and/or mental health care professionals. Considerable relevant medical and psychological data is provided by these professionals to our clinic. Experienced child and adolescent psychiatrists conduct semi-structured interviews with parents and children, following the DSM 5 criteria for ARFID diagnosis [1]. These interviews further assess the subtype of ARFID according to the criteria of the DSM 5 [1]. We do not use standardized interviews for the diagnosis of ARFID because none of these tools has been translated to Hebrew or validated in Israeli populations.

Comorbid psychiatric diagnoses were assessed by the psychiatrists with interviewing the parents and children using the Structured Clinical Interview for DSM-IV Axis I Disorders (SCID)—Patient Edition [36], adapted for DSM-5 [1]. Diagnosis of comorbid ADHD was obtained using the ADHD module of the Schedule for Affective Disorders and Schizophrenia for school-age children—present and lifetime version (K-SADS-PL) [43]. In this study we used the SCID-I/P Version 2.0 [36] rather than the K-SADS-PL [43] to diagnose comorbid psychiatric disorders because although we were investigating children, the parents were our main interviewees (children were evaluated with their parents; there is no ADHD module in the SCID-I/P Version 2.0). We have used this assessment method in our previous study in children with ARFID [37].

Clinical nutritionists assess nutritional intake with 24-hours dietary recall, estimating daily caloric intake and food variety, according to Bryant-Waugh’s checklist [44]. This assessment systematically checks the child’s eating and feeding habits and is based on the DSM-5 Criterion A of ARFID [1], i.e., avoidance of food (variety and amount) not related to dieting/body image disturbances, that interferes with the child’s medical condition, requiring nutritional supplementation, and with his/her psychosocial functioning, and social life. All diagnoses are confirmed in multi-professional clinical team meetings. Only children with confirmed ARFID diagnoses according to these procedures have been included in this retrospective study. This design is similar to that used in a previous study of our group in children with ARFID [45].

The treatment as usual (TAU) in our facility is based on the principles of Thomas et al.‘s [21] cognitive-behavioral therapy for avoidant/restrictive food intake disorder. It can be delivered in either a group or an individual format. The group intervention starts with initial art therapy work to increase motivation and reduce anxiety, followed by graded exposure (the taillight model) and cognitive interventions, using art-therapy techniques. There is also a psychoeducational group for parents, and regular nutritional follow-up. The individual format uses similar techniques.

Children are referred to one of the two formats according to clinical judgment whether they fit more a group or an individual format. The type of ARFID per se does not direct the specific treatment the child will receive. However, as children with the sensory sensitivity ARFID subtype may be often diagnosed with comorbid ADHD, they might not benefit from group interventions. By contrast, children with the lack of interest subtype may often show comorbid social anxiety disorder, hence benefitting considerably from a group intervention. By the same token, children with the sensory sensitive subtype may require medications for their ADHD, whereas children with lack of interest and aversion to food may benefit from specific serotonin reuptake inhibitors (SSRIs) for the comorbid anxiety disorders.

### Demographic and clinical data

The following demographic and clinical data were collected from the patients’ computerized medical records: (1) age and gender, (2) ARFID subtype, (3) medical history, (4) psychiatric comorbidity, (5) prior medications, (6) type and dosage of AAP administered, (7) adjunctive psychotropic medications, (8) weight/weight%, height/height%, and BMI/BMI%, based on the National Center for Health Statistics (https://www.cdc.gov/growthcharts/cdc_charts.htm). This data was collected from the patients’ records every three months; (9) nature and timing of adverse events as documented in the patients’ records.

### Clinical global impression scale

The severity of ARFID symptoms was assessed retrospectively using the Clinical Global Impression Severity Scale (CGI-S) [46], a seven-point scale ranging from one (normal) to seven (most severely ill). Two child and adolescent psychiatrists (M.M., D.S.) independently evaluated the CGI-S based on the patients’ computerized medical records. One psychiatrist (M.M), aware of the patients’ names but not present during AAP treatment, collaborated with another psychiatrist (D.S.), blind to the patients’ names but present during AAP augmentation. The severity of the symptoms was based on the severity of avoidance and restriction, anxiety around eating, problems in social/school functioning, conflicts with parents over eating, and comorbid depressive and anxiety symptoms. Assessments of CGI symptom severity were conducted at initial evaluation, start of AAP treatment, and termination of AAP treatment, with additional assessments performed if medications changed. The retrospective completion of the CGI based on chart reports was successfully used in previous pediatric studies [47, 48]. Inter-rater reliability between the two psychiatrists was high (*r* = 0.87, *p* < 0.001).

Assessment of adverse effects: Two psychiatrists (M.M, D.S.) examined independently the computerized medical files of all participants from the beginning to the end of the study. The severity of the adverse effects was rated according to the descriptions in the computerized files by the treating psychiatrists.

### Statistical analysis

Descriptive statistics were produced using frequencies (n; %) for categorical variables (e.g. sex) and means with standard deviations for continuous variables (e.g. age). To assess change in BMI, weight, and height over time, we used Friedman’s tests. When significant results were obtained, Wilcoxon test for pairwise differences was conducted. To prevent familywise alpha inflation, Bonferroni post-hoc corrections were applied to the p-values of the pairwise contrasts. Statistical comparisons were conducted using raw data, rather than percentiles, to optimize the statistical power of the calculations due to the larger sensitivity of the raw data. Visualizations of these effects were demonstrated using Box-Plots. P-value for all analyses was 5%. Data were analyzed using SPSS version 27.

## Results

Twenty-one children (11 boys and 10 girls) aged 10.54 ± 3.21 years at diagnosis of ARFID (range 6.2–17.2 years), were included in the study. The demographic and clinical characteristics of the participants are summarized in Table 1. The duration of follow-up was 18 months. The mean period between diagnosis and treatment initiation was 6.30 ± 0.75 months (range 0-33.74 months). No differences were found in the demographic and clinical variables assessed between the 14 children starting with an AAP in our facility, and the 7 children receiving AAPs in another facility before admitted to our setting because of failure to gain weight. Because of these findings we decided to combine the two groups.

**Table 1:** Sociodemographic and clinical characteristics of the study sample

	Sex	Age at diagnosis of ARFID (years)	Percentile at diagnosis			Age at the beginning of treatment (years)	ARFID subtype	Atypical Antipsychotic	Other psychotropic medications	Comorbidity
			BMI%	Weight%	Height%					
1	F	7.64	2.0	0.3	1.7	9.24	Sensory sensitivity	Risperidone	Fluoxetine	Social anxiety disorder, Separation anxiety disorder, School refusal
2	F	11.51	11.0	0.1	0.1	11.72	Lack of interest	Risperidone		
3	M	6.64	4.0	47.6	82.2	9.45	Lack of interest	Risperidone		
4	M	9.77	0.2	0.2	12.3	10.13	Lack of interest	Risperidone		ADHD
5	M	11.11	7.0	3.3	9.7	11.38	Lack of interest	Risperidone		
6	M	6.84	30.0	29.4	37.6	8.22	Lack of interest	Risperidone	Fluoxetine	Social anxiety disorder, Separation anxiety disorder, School refusal
7	F	11.21	2.0	2.5	18.1	12.70	Lack of interest	Risperidone	Atomoxetine	ADHD
8	M	6.78	41.0	3.5	1.3	6.78	Sensory sensitivity	Risperidone		
9	F	11.30	0.1	0.1	1.0	11.30	Sensory sensitivity	Risperidone	Fluoxetine, Somatropin (growth hormone)	Separation anxiety disorder, ADHD
10	F	12.94	0.5	0.3	0.9	12.94	Combined	Risperidone	Somatropin (growth hormone) Fluoxetine	Separation anxiety disorder, Depressive episode
11	M	11.56	0.1	0.2	4.0	12.96	Combined	Risperidone	Somatropin (growth hormone)	
12	M	12.19	23.0	42.7	77.7	12.90	Lack of interest	Risperidone		ADHD
13	F	12.14	3.0	1.2	6.7	13.28	Sensory sensitivity	Risperidone	Papaverine, Escitalopram	depressive episode ADHD
14	M	10.27	1.0	33.1	93.1	10.27	Combined	Risperidone	Methylphenidate, Fluoxetine, Amitriptyline Atomoxetine, Levomepromazine	ADHD
15	M	6.22	20.0	31.6	53.1	6.56	Combined	Olanzapine		ADHD
16	F	7.97	1.0	0.1	0.8	7.97	Lack of interest	Risperidone	Atomoxetine, Dexmethylphenidate	ADHD, Motor and vocal tics
17	M	9.98	7.0	37.8	83.5	10.03	Sensory sensitivity	Risperidone	Fluoxetine	Atypical anxiety disorder, ODD, Dysthymia
18	F	6.68	49.0	32.6	24.3	6.68	Sensory sensitivity	Risperidone		Separation anxiety disorder
19	F	16.81	0.1	0.1	18.4	17.61	Sensory sensitivity	Risperidone		
20	F	14.51	0.3	0.5	30.9	15.14	Sensory sensitivity	Risperidone	Fluoxetine	Atypical anxiety disorder
21	M	17.23	0.1	0.1	3.4	17.23	Lack of interest	Risperidone	Sertraline	Separation anxiety disorder

Note: ADHD: Attention deficit anxiety disorder, ODD: Oppositional defiant disorder

Twenty children were started with risperidone, and one with olanzapine. This patient came from the group of 7 patients already admitted with AAP treatment, and we have no information as to why he was started with olanzapine. Four children had to be switched to olanzapine, three because of lack of improvement with risperidone and one because of developing fatigue.

Fifteen children (71%) had evidence of comorbid psychiatric disorders, of them, seven (33%) were diagnosed with more than one comorbid disorder. Anxiety disorders and ADHD were diagnosed in 8 children each. Eleven (52%) children received additional psychotropic medications, with the majority of them receiving adjunctive SSRIs because of depressive/anxiety symptoms (*n* = 9, 42%) (Table 1). Three patients were treated for ADHD. Another three patients received somatomedin (growth hormone), All were pre-adolescents, and their endocrinologists started with the medication only when they showed consistent weight gain. One patient was treated with levomepromazine when required because of occasional irritability. Additional SSRI was prescribed to 5/9 children with the lack of interest subtype, 2/8 children with the sensory sensitivity type, and 2/4 children with the combined type. In all cases the additional psychotropic medications were given when the patient was already treated with the AAP and gaining weight, most of them because of persisting depressive or anxiety symptoms.

Of the 21 patients in the study, 8 were diagnosed with the sensory sensitivity subtype, 9 with the lack of interest type, and 4 with the combined presentation see Table 1). No patient in this study was diagnosed with fear of aversive consequences subtype. The female/male ratios in the three subtypes were 6/2 for sensory sensitivity, 3/6 for lack of interest, and 1/3 for the combined type. Comorbid attention deficit hyperactivity disorder (ADHD) was diagnosed in 4/8 patients with sensory sensitivity, 2/9 patients with lack of interest, and 2/4 of the combined category. Any anxiety disorder was diagnosed in 2/8 patients with sensory sensitivity, 5/9 patients with lack of interest, and 1/4 of the combined category. Additional SSRIs were prescribed to 5/9 children with the lack of interest subtype, 2/8 children with the sensitivity type, and 2/4 children with the combined type.

### Change over time in weight/weight%, height/height%, and BMI/BMI%

No significant differences were observed between the time of diagnosis and treatment initiation in mean weight, height, and BMI, while their respective percentiles were reduced. A significant increase was noted in weight (9.66 ± 9.24 kg; Friedman’s χ2(7) = 59.88, *p* < 0.001), height (10.23 ± 11.54 cm; Friedman’s χ2(7) = 55.43, *p* < 0.001), and BMI (2.55 ± 1.53 kg/m2; Friedman’s χ2(7) = 39.87, *p* < 0.001) during the 18-month follow-up period (Table 2).

**Table 2:** Changes in weight, height and BMI over time

Outcome	Diagnosis	Treatment beginning	3 months	6 months	9 months	12 months	15 months	18 months	*p*-value
Weight **Mean** **(± SD)**	25.91 (7.05)	27.02 (6.86)	29.28 (7.3)	31.72 (9.05)	32.84 (9.2)	34.93 (9.23)	35.51 (9.06)	36.68 (9.94)	< 0.001
Weight% **(± SD)**	12.69 (17.59)	8.44 (12.51)	15.12 (17.18)	21.9 (24.23)	20.60 (22.20)	26.6 (28.22)	27.45 (30.17)	31.80 (31.54)	
Height **Mean** **(± SD)**	133.43 (15.56)	135.62 (13.44)	138.17 (14.51)	139.58 (14.38)	142.14 (13.52)	144.04 (13.49)	144.81 (12.74)	145.85 (13.12)	< 0.001
Height % **(± SD)**	24.40 (14.07)	19.90 (27.70)	23.09 (29.27)	24.05 (30.37)	24.18 (29.37)	27.28 (31.81)	27.90 (26.60)	30.82 (35.78)	
BMI **Mean** **(± SD)**	14.18 (1.27)	14.63 (1.53)	15.30 (1.70)	16.12 (1.92)	16.10 (2.15)	16.76 (2.15)	16.85 (2.13)	17.18 (2.5)	< 0.001
BMI (%)	9.80 (7.03)	8.47 (5.27)	11.34 (3.70)	18.94 (3.79)	18.17 (3.87)	20.00 (4.03)	21.80 (4.35)	24.82 (4.48)	

Note: BMI: body mass index; %: percentile

Specifically, mean weight change in the first three months of treatment was 2.26 kg, and 4.70 kg in the first six months. Subsequently, mean weight gain in the following six months was 3.21 kg, and 1.75 kg between 12 and 18 months, indicating the greatest weight gain occurred within the first six months of AAP treatment. Weight percentile increased from 12.69% at diagnosis to 15.12% at treatment initiation and further to 31.8% at 18-month follow-up.

**Fig. 1:**
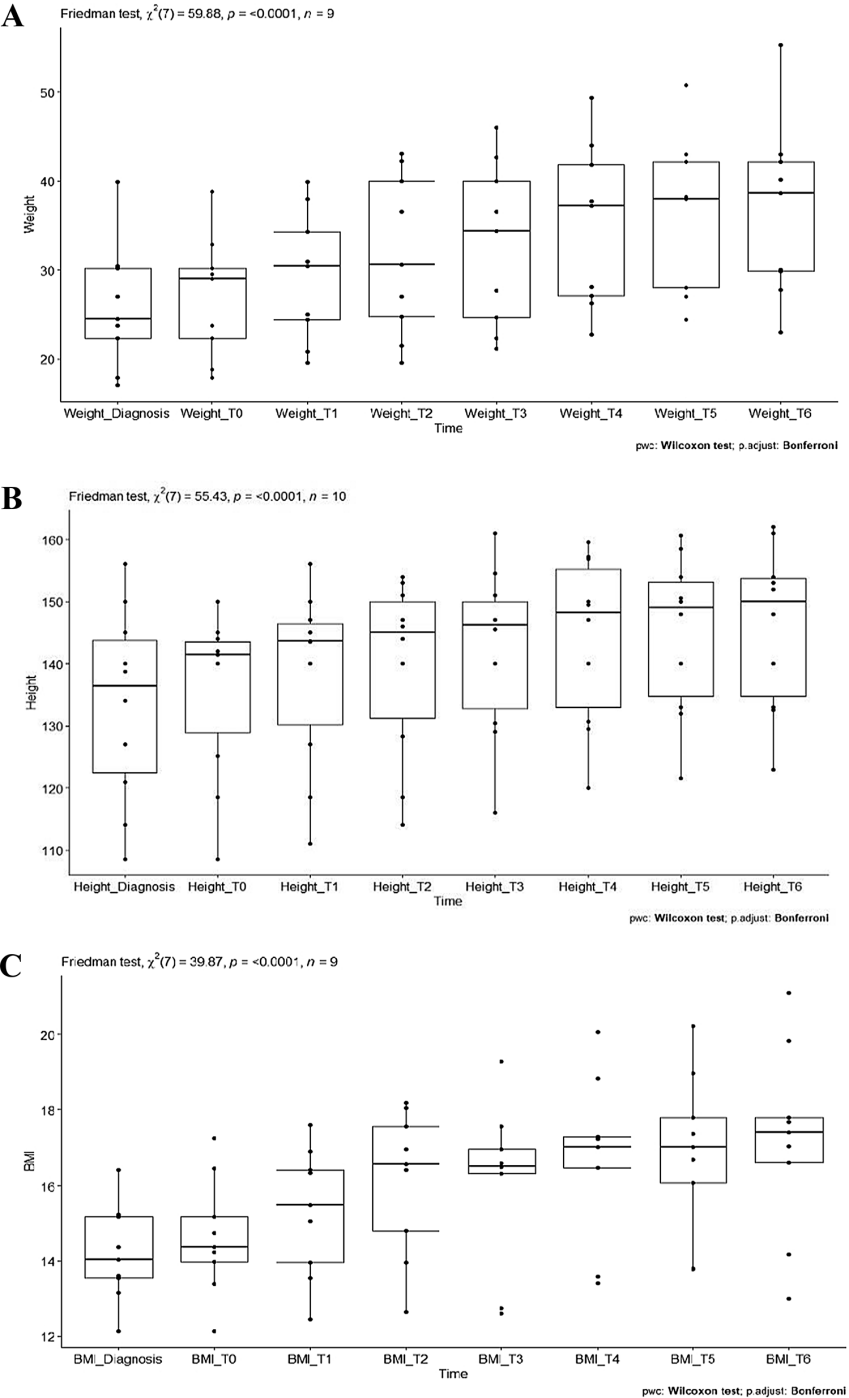
Change of weight, height, and BMI over 18 month period with a typical. Antipsychotics treatment. **a**: Change of weight over time. **b**: change of height over time. **c**: change of BMI over time. Note: BMI: body mass index; TO: beginning of treatment; T1: 3 months; T2: 6 months; T3: 9 months; T4: 12 months; T5: 15 months; T6: 18

The mean height change in the first three months of treatment was 2.25 cm, and 3.96 cm in the first six months. The subsequent mean height gain in the following six months was 4.46 cm, with an additional gain of 1.82 cm between months 12 and 18, suggesting that linear growth was most prominent in the first 12 months of treatment, particularly between months 6 and 12. Height percentile decreased from 24.4% at diagnosis to 19.9% at AAP initiation but increased to 30.8% at 18-month follow-up. Moreover, a consistent increase was predominantly observed at every three-month interval from 3 to 18 months’ follow-up for weight (except for no increase from 3 to 6 months follow-up), height, and BMI, with a similar trend found for weight, height, and BMI percentiles (Table 2; Fig. 1).

Risperidone was started in low doses, i.e., 0.2 mg/day, and raised slowly. i.e., increments of 0.2 mg/day every 4–6 weeks. The maximal dose of risperidone was usually between 0.6 and 0.8 mg/day; nonetheless, the risperidone dosage required in three children and the three adolescents included in the study was between 1 and 1.5 mg/day. With respect to olanzapine, we started with 1.25 mg/day, and the maximal dose was 5 mg/day.

## Effects of potential confounders

### Sex

When measuring the effect of sex as a possible confounder on the results (Table 3), we found that weight increased significantly over time for both males (Friedman’s χ2(7) = 33.50, *p* < 0.001) and females (Friedman’s χ2(7) = 20.2, *p* = 0.005). By contrast, height increased significantly over time only for males (Friedman’s χ^2^(7) = 35.39, *p* < 0.001) but a trend was shown for females (Friedman’s χ^2^(7) = 13.79, *p* = 0.06). Last, BMI increase over time was significant for males (Friedman’s χ2(7) = 26.29, *p* < 0.001) but not for females (Friedman’s χ2(7) = 10.65, *p* = 0.15).

**Table 3:** Change over time in mean (SD), weight, height, and BMI according to sex

Outcome	Group	Diagnosis	T0	T1	T2	T3	T4	T5	T6	*p*-Value
Weight	Male	28.6 (8.14)	29.76 (7.63)	32.7 (7.57)	36.69 (8.86)	37.52 (9.18)	39.9 (8.39)	40.4 (8.33)	41.84 (9.12)	< 0.001
	Female	22.63 (5.05)	24.03 (5.35)	25 (5.72)	25 (5.5)	26.77 (6.84)	28.93 (7.79)	29.87 (7.33)	30.33 (8.88)	0.005
Height	Male	136.77 (17.37)	138.52 (14.97)	142.12 (15.86)	144.17 (15.12)	146.08 (15.65)	148.45 (14.74)	148.77 (14.2)	150.17 (14.3)	< 0.001
	Female	130.9 (14.65)	133.3 (12.84)	134 (13.54)	134.17 (13.71)	138.17 (8.4)	139.67 (10)	141.17 (8.81)	141.67 (9.61)	0.06
BMI	Male	14.62 (1.19)	15.44 (1.38)	16.15 (1.53)	17.45 (0.7)	17.36 (1.15)	18.08 (1.33)	18.2 (1.41)	18.6 (1.82)	< 0.001
	Female	13.11 (0.95)	13.43 (1.31)	13.82 (1.31)	13.8 (1.08)	13.95 (2.21)	14.67 (2.02)	14.86 (1.85)	14.86 (2.28)	0.15

Note: BMI: body mass index; T0: beginning of treatment; T1: 3 months; T2: 6 months; T3: 9 months; T4: 12 months; T5: 15 months; T6: 18

### Age

No differences were found in weight, height and BMI when the sample was split into two groups according to the median age (11 years). The increase in both age groups was significant for all these three parameters (results not shown).

### Baseline BMI%

The sample was divided into two groups based on the median BMI% at treatment initiation (Table 4). Weight significantly increased over time for both low BMI% (Friedman’s χ2(7) = 17.72, *p* = 0.006) and high BMI% groups (Friedman’s χ2(7) = 12.81, *p* < 0.001). Notably, individuals with high baseline BMI% gained more weight in the first six months compared to those with low BMI (7.11 kg vs. 3.48 kg). Conversely, those with low baseline BMI% showed greater weight gain between 6 and 12 months (high BMI% 1.69 kg vs. low BMI 3.99 kg) and 12–18 months (high BMI% 0.23 kg vs. low BMI% 2.46 kg).

**Table 4:** Change over time of weight and height according to low and high BMI percentile (BMI%)

Outcome	Group	Diagnosis	T0	T1	T2	T3	T4	T5	T6	*p*-Value
Weight	Low BMI%	25.73 (8.6)	26.25 (8.06)	28.43 (8.01)	29.73 (8.99)	31.37 (9.48)	33.72 (9.68)	34.57 (9.78)	36.18 (11.31)	< 0.001
	High BMI%	26.27 (3.68)	28.57 (4.57)	30.97 (6.81)	35.68 (9.49)	35.8 (9.71)	37.37 (9.65)	37.4 (9.02)	37.67 (8.55)	0.006
Height	Low BMI%	130.04 (17.23)	132.96 (15.55)	134.96 (16.34)	135.97 (15.59)	138.49 (13.61)	140.51 (13.66)	141.93 (13.2)	143.36 (14.00)	< 0.001
	High BMI%	141.33 (8.08)	141.83 (2.02)	145.67 (5.13)	148 (7.00)	150.67 (10.5)	152.27 (10.69)	151.53 (10.52)	151.67 (10.69)	0.10

Note: BMI: body mass index; T0: beginning of treatment; T1: 3 months; T2: 6 months; T3: 9 months; T4: 12 months; T5: 15 months; T6: 18 months

Second, mean height increased significantly over time only for patients with low BMI% (Friedman’s χ^2^(7) = 19.8, *p* = 0.006; 12.66 cm change) but not for patients with high BMI% (Friedman’s χ^2^(7) = 12, *p* = 0.10; 10.33 cm change). Nonetheless, it is of note that the difference in linear growth between the two groups (mean of 2.33 cm over a period of 18 months), does not seem to be meaningful from a clinical consideration.

Although no statistical analyses could be performed because of the small numbers in each category, there seemed to be no differences in weight, height, BMI, and their percentiles between the 14 patients stating AAP in our setting and those admitted with AAPs, and between the different ARFID subtypes.

### Side effects

Overall, seven patients (33.4%) had evidence of side effects, mostly mild, including headaches (*n* = 1), nighttime awakening (*n* = 1), fatigue (*n* = 4), tics (*n* = 2), irritability (*n* = 2) secondary enuresis (*n* = 1), and increased hunger (*n* = 2).

### Treatment drop out

Twenty-one patients initially started AAP treatment, with 19 continuing after 3 months, 16 after 6 months, 13 after 12 months, 8 after 15 months, and 7 after 18 months. Reasons for dropouts included loss of follow-up due to the COVID-19 pandemic (*n* = 3), lack of improvement (*n* = 4), and termination of treatment due to symptomatic improvement and weight gain (*n* = 7). No patients discontinued AAP medications due to adverse effects.

### Clinical improvement

The mean CGI-S score at time of diagnosis was 5.24 ± 1.09. No change was shown from the time of diagnosis to the start of AAP treatment (5.62 ± 0.80; *p* = 0.05). By contrast, we found a significant improvement from the beginning of treatment to follow-up (defined as the time point of termination of treatment) in severity of ARFID (5.62 ± 0.80 vs. 3.09 ± 1.17, respectively, *t* = 3.25, df = 20, *p* = 0.003). Last, significant clinical improvement, defined as minimal illness (CGI-S = 2) or no illness (CGI-S = 1) at follow-up was, found in 14/21 patients (66.6%).

## Discussion

In line with our first hypothesis, children with severe ARFID, unresponsive to psychological interventions (TAU) for at least six months in our center (*n* = 14) or those receiving AAPs in prior settings because of lack weight increase (*n* = 7), showed a significant improvements in weight/weight%, height/height%, and BMI/BMI% after 3–18 months of AAP treatment. In line with our second hypothesis, AAP treatment was safe and well tolerated in our study sample. The most common side effects were drowsiness and sedation, and none of the participants required early discontinuation because of adverse effects.

Weight/weight% increased mostly within the first 12 months of AAP treatment, with smaller improvements thereafter probably due to fewer participants remaining in the study. Alternatively, the weight increasing effect of the AAP might have decreased over time. In keeping with this contention, prior studies showed that children previously not exposed to antipsychotics were particularly vulnerable to weight gain, occurring mostly within the first few months of treatment [42, 43], and likely stabilizing thereafter. Nonetheless, in contrast to our children with ARFID-related malnutrition, these children have been of normal weight, likely not malnourished, receiving antipsychotic medications because of schizophrenic-spectrum, affective-spectrum, autism spectrum, or behavioral disorders.

Height/height% showed significant increases mainly between 12 and 18 months, likely following the increase in weight/weight%. Notably, height percentile decreased from 24.4% at diagnosis to 19.9% at treatment initiation but increased to 30.8% at 18 months’ follow-up. Unfortunately, we do not have data as to the change in weight and height in the 159 patients in our clinic not treated with AAPs.

Dunbar et al. [49] reviewed five studies administering risperidone (mean dose 1.3.

mg/day) to normal-weight children (mean age 10.2 ± 2.4 years) with disruptive disorders. In a subset of 350 children assessed for height over 12 months, it increased by 6.3 cm. This increase is smaller than in our sample (mean age 10.54 ± 3.2 years) − 10.23 ± 11.54 cm, possibly due to the shorter follow-up period (12 vs. 18 months) and risperidone’s potentially greater effect in malnourished children. Both studies highlight no significant growth failure with risperidone [49].

Emerging findings indicate that youth are especially vulnerable to antipsychotic-induced weight gain [50], although other studies do not support this contention [51]. Our patients and their parents have often indicated considerable increase in appetite following AAP treatment, potentially directly related to the influence of the AAPs [43]. In addition, weight gain in our sample could have been associated with increased appetite related to the overall improvement in the children’s emotional condition, as indicated in the significant reduction in the CGI scores, including in anxiety and depression [52]. The improvement of the CGI-S at the end of treatment is of importance, as studies in anorexia nervosa have shown that improvement of eating disorders (ED) symptoms occurs alongside an improvement in comorbid symptoms, including anxiety and depression [53].

Animal models employing growth-inhibiting conditions, such as malnutrition, show functional changes in the growth plates which inhibit cellular proliferation and growth [54]. When these conditions resolve, growth plates show accelerated proliferation resulting in catch-up linear growth [55]. As the mean height of healthy children is assumed to be around the 50th percentile, the height percentile of our cohort (24.40 ± 14.07%), is likely compromised.

Indeed, restrictive EDs, notably anorexia nervosa, often lead to growth deceleration and stunting [55, 56], potentially affecting adult height if occurring during crucial growth periods. Nutritional rehabilitation can accelerate linear growth [56, 57], but complete catch-up growth may not always occur, impacting adult height [58]. While this is well-documented in anorexia nervosa, emphasizing the need for adequate nutritional rehabilitation [55, 56], growth deceleration and correction post-treatment remain unexplored in ARFID. Our study observed an increase in height percentile from 24.4% at diagnosis to 30.8% at 18 months’ follow-up, indicating the importance of early caloric intake augmentation, complemented with AAP administration. However, complete correction of the compromised height of our patients (i.e., achieving the 50th height percentile) has not been achieved at 18 months’ follow-up. Other studies have shown that catch-up growth in pre-menarcheal girls with restrictive EDs (although not ARFID) may not be achieved until 2–4 years from treatment initiation [59].

As shown in our findings, a time lag has been noted between the increase in weight/weight% and height/height%. This finding seems plausible, as in young people with malnutrition, deviations or percentile drops in weight occur first, followed by deviations in height [60]. Indeed, studies assessing malnourished children in impoverished countries, e.g. Bangladesh [61], or Nepal [62], have found a similar time lag between weight gain and height gain. One study in Nepal [63] emphasizes that in the majority of malnourished children, linear growth does not begin until they have achieved at least 85% of their expected weight-for-length. In addition, the weight percentile of our children on admission (12.69 ± 17.59%) has been more compromised than their height percentile (24.40 ± 14.07%), so that essential physiological disturbances related to malnutrition had to be corrected before the acceleration of linear growth.

In our study, we seem not to find different trends in the increase in weight, height, BMI and their percentages among the different ARFID subtypes requiring AAPs, although the number of the patients in the different groups is too small to draw any statistical inferences. The demographic and clinical variables in our subtypes show findings that are similar to other studies, i.e., a greater number of males and comorbid ADHD in the sensory sensitivity subtype, and a greater number of females and comorbid anxiety disorders in the lack of interest subtype [6]. While usually the lack of interest subtype is associated with the highest risk of weight reduction and low weight, and the sensory sensitivity subtype with the lowest [5, 6], the patients in this study in all groups are considered severely ill if requiring not only AAPs, but also additional SSRIs in more than a third. The direct influence of SSRIs on weight is variable (although less than that of AAPs), with some medications showing greater weight increase (e.g., escitalopram) and others less weight increase (e.g., fluoxetine, given to 7/9 patients receiving SSRIs). Nonetheless, SSRIs may increase appetite, and hence weight, due their anxiolytic and antidepressant properties, and in our sample, more children with the lack of interest subtype received SSRIs than the sensory sensitive subtype. It is of note that none of the children was treated with cyproheptadine, an antihistamine anti-serotonergic agent appetite stimulant. This medication is quite expensive for prolonged use, and in Israel, the health services do not support financially the administration of cyproheptadine in ARFID.

### Factors influencing the potential of AAPs to improve food consumption and weight

Looking at possible confounders of our findings, we found that that whereas weight increased both for boys and girls, height increased significantly only for boys, although a clinical improvement was also noted for girls. As the mean age at entry was 12.05 ± 3.50 for girls and 10.58 ± 3.06 for boys, and taking into consideration the earlier pubertal development in girls vs. boys [64], some girls in our study might have been already closer to the final adult height. Another possible confounder is baseline BMI%. It showed no effect on weight gain at the study’s end but influenced weight changes over time. Children with higher baseline BMI% gained more weight in the first six months, while those with lower BMI% gained more in the second six months, suggesting that physiological corrections are needed before significant weight gain occurs in lower BMI% children. Studies in children with psychiatric disorders have found that lower-weight individuals are more likely to gain weight with AAP treatment [34, 65]. Additionally, children with higher baseline BMI% have not grown in height compared to those with lower BMI%; however due to our study’s small sample size, no conclusions can be drawn.

Our findings are only preliminary and should be handled with caution because of several important limitations. (1) Our study is a retrospective open naturalistic follow-up study of a relatively small cohort, lacking a control group of children with ARFID not receiving AAPs (we have not analyzed the respective data of the 159 children treated in our facility and not receiving AAPs). (2) The group includes two different populations, those stating with the AAP and those already admitted with AAP treatment. Nonetheless, in both conditions the AAP has been given because of failure to gain weight. (3) other concomitant medications have been administered to more than half of the patients, with some of them having definite weight increasing properties (e.g., levomepromazine and somatomedin). Nonetheless, these medications have been given after the administration of the AAP, when the increase in weight is mostly present. For example, the treating endocrinology specialists have been ready to administer somatomedin, only after the child has shown definite improvement in eating and weight. (4) we have not used standardized tools for the diagnosis of ARFID. Nonetheless, the diagnosis of the disorder in our sample relies on independent psychiatric and structured systematic nutritional evaluations, and on relevant medical and psychologic data from previous treatment providers. (5) we have not included in our analysis physical data such as pubertal stage or menstruation. However, most of our sample is pre-pubertal. Similarly, we have not taken into account the effect of age change on the linear growth of our participants during follow-up. (6) we have not examined systemically some of the side effects of AAPs such as disturbances in lipid profile and hyperprolactinemia, because of the reluctance of many young participants in clinical settings to undergo blood testing. (7) assessment of improvement has been conducted retrospectively with the CGI-S, based on a well-documented chart review of each visit. It has not been done with measures of ARFID symptomatology because none of these tools has been translated to Hebrew or validated in Israeli populations. (8) TAU has been decided on clinical grounds and could be changed during the course of AAP treatment if required. (9) Fourteen patients have not completed the 18-month follow-up period. However, in half of the sample it is because of symptomatic improvement, and there has been no premature termination because of adverse effects.

The study holds several important advantages. Although small, our sample is likely the largest group assessed yet. Psychiatric follow-up was structured, interviewing both children and parents, to ensure medication adherence, although pill counting was not conducted in this clinical setting. Throughout AAP treatment, all patients and parents continued to receive personalized psychological interventions and nutritional counseling.

In summary, our study demonstrates that low doses of AAPs, primarily risperidone, can effectively increase weight and height in young individuals with ARFID who fail to increase their weight before AAP treatment, with minimal side effects. The observed differences in weight and height percentile increases suggest that AAP augmentation should be continued for at least 12 months, with regular monitoring of physical and laboratory parameters.

If the addition of AAPs in children with ARFID not improving without these medications would prove beneficial in large scale open studies, future studies should be controlled and randomized. They should include patients with different types of ARFID, including adults, relating to the influence of relevant comorbid psychiatric disorders, with a longer follow-up duration, standardization of other medications and of concomitant psychological interventions, and assessing different AAPs.

## Electronic supplementary material

Below is the link to the electronic supplementary material.

Supplementary Material 1

## Data Availability

No datasets were generated or analysed during the current study.
