# Supplementary material for: The use of atypical antipsychotic medications in the treatment of children and adolescents with avoidant/restrictive food intake disorder
Source: Eur Child Adolesc Psychiatry. 2025 Apr 24;34(10):3083–95. doi: 10.1007/s00787-025-02713-w (PMC12592295; doi:10.1007/s00787-025-02713-w)
Supplement: Supplementary file 1 — Supplementary Material 1 [file 787_2025_2713_MOESM1_ESM.docx]

***Treatment protocol in Child and Adolescent Eating Disorder Outpatient Clinic at the Pediatric Psychosomatic Department, Safra Children's Hospital, Sheba Medical Center***

Children treated in our pediatric ED clinic because of ARFID may receive two possible treatment as usual (TAU) protocols. One protocol is conducted in a group format, where around six children of the same age range and gender receive treatment from two therapists: an expression therapist (art therapy, movement therapy, or drama therapy) and a clinical psychologist/social worker. This protocol uses expression-therapy techniques to reduce ARFID-related anxieties employing a behavioral "traffic-light" graded systematic exposure, alongside CBT techniques adapted for children to treat faulty cognition. At the same time, the children are regularly followed by a clinical nutritionist. The children's parents attend regular psychoeducational groups carried out by a therapist (psychologist, social worker, or expression therapist) and a clinical nutritionist. The children groups are carried out once weekly and the parent groups every two weeks.

The other protocol uses an individual format led by a therapist and a clinical nutritionist. Using the same treatment protocol as in the group format, the therapist meets with the child once weekly and with the parents every two weeks, while the clinical nutritionist meets with the child and parents every two weeks. The choice of the suitable treatment for each child is based on clinical considerations.
